# Effect of NAD^+^ boosting on kidney ischemia-reperfusion injury

**DOI:** 10.1371/journal.pone.0252554

**Published:** 2021-06-01

**Authors:** Marya Morevati, Søren Egstrand, Anders Nordholm, Maria L. Mace, Claus B. Andersen, Rouzbeh Salmani, Klaus Olgaard, Ewa Lewin

**Affiliations:** 1 Nephrological Department P, Rigshospitalet, University of Copenhagen, Copenhagen, Denmark; 2 Nephrological Department B, Herlev Hospital, University of Copenhagen, Copenhagen, Denmark; 3 Department of Forensic Pathology, Rigshospitalet, University of Copenhagen, Copenhagen, Denmark; 4 Department of Pathology, Roskilde Hospital, University of Copenhagen, Roskilde, Denmark; Universidade de Sao Paulo, BRAZIL

## Abstract

Acute kidney injury (AKI) is associated with a very high mortality and an increased risk for progression to chronic kidney disease (CKD). Ischemia-reperfusion injury (IRI) is a model for AKI, which results in tubular damage, dysfunction of the mitochondria and autophagy, and in decreased cellular nicotinamide adenine dinucleotide (NAD^+^) with progressing fibrosis resulting in CKD. NAD^+^ is a co-enzyme for several proteins, including the NAD^+^ dependent sirtuins. NAD^+^ augmentation, e.g. by use of its precursor nicotinamide riboside (NR), improves mitochondrial homeostasis and organismal metabolism in many species. In the present investigation the effects of prophylactic administration of NR on IRI-induced AKI were studied in the rat. Bilateral IRI reduced kidney tissue NAD^+^, caused tubular damage, reduced α-Klotho (klotho), and altered autophagy flux. AKI initiated progression to CKD, as shown by induced profibrotic Periostin (*postn*) and Inhibin subunit beta-A, (*activin A* / *Inhba*), both 24 hours and 14 days after surgery. NR restored tissue NAD^+^ to that of the sham group, increased autophagy (reduced p62) and sirtuin1 (*Sirt1*) but did not ameliorate renal tubular damage and profibrotic genes in the 24 hours and 14 days IRI models. AKI induced NAD^+^ depletion and impaired autophagy, while augmentation of NAD^+^ by NR restored tissue NAD^+^ and increased autophagy, possibly serving as a protective response. However, prophylactic administration of NR did not ameliorate tubular damage of the IRI rats nor rescued the initiation of fibrosis in the long-term AKI to CKD model, which is a pivotal event in CKD pathogenesis.

## Introduction

Acute kidney injury (AKI) is a common syndrome affecting 5–7% of all hospitalized patients [1]. It is associated with a significant increase in the length of hospital stay, in a very high mortality and often increased risk for progression to chronic kidney disease (CKD) and development of end-stage renal disease (ESRD) [2–5]. Tubular-interstitial fibrosis is the major pathway of progression to ESRD regardless of the diverse origin of kidney pathology, such as glomerular, vascular, or tubular [6–8]. Ischemia-reperfusion injury (IRI) of the kidney is a leading cause of clinical AKI, which frequently occurs in patients with sepsis, patients subjected to major surgery.

Renal IRI is characterized by the limited blood supply to the kidney, followed by restoration of blood flow and re-oxygenation of the tissue. AKI severity and the subsequent AKI to CKD progression dependent upon the duration of ischemia and clinical factors, including medical condition, advanced age, and repeated episodes of kidney injury [9–12]. The pathophysiology of IRI includes tubular epithelial cell injury, endothelial injury, tubular cell death by apoptosis and necrosis signaling, inflammation, production of reactive oxygen species (ROS), and activation of autophagy [13–15]. Proximal tubular epithelial cells reabsorb the bulk of the ultrafiltrate and consume huge amounts of energy [16]. The kidney is the organ with second highest number of mitochondria after the heart [17,18]. Mitochondrial dysfunction leads to decreased ATP production in the mitochondria, dysfunction of sodium-potassium pumps, detachment of ribosomes, elevated mitochondrial ROS generation, increased intracellular calcium, and activation of membrane phospholipids proteases [19–21]. A decline in antioxidants such as catalase (Cat), mitochondrial super-oxidase dismutase 2 (SOD2), and glutathione peroxidase might contribute to the pathophysiology of IRI [22].

Nicotinamide adenine dinucleotide (NAD^+^) has been shown to exert an essential function in redox reactions in metabolic processes and mitochondrial oxidative phosphorylation [23,24]. Additionally, several intracellular proteins, including poly-ADP-ribose polymerases (PARPs), sirtuins and cyclic ADP-ribose synthetases, such as CD38, consume NAD^+^ in their enzymatic reactions [25,26]. The sirtuin family is an NAD^+^ dependent histone deacetylase family of lysine [27–29]. In the kidney, sirtuin 1 (SIRT1) is highly expressed in the proximal tubules, where it has a protective effect on AKI [30,31].

NAD^+^ homeostasis correlates intimately with autophagy, a conserved pathway for clearance of damaged organelles [32,33]. Autophagy is important for kidney homeostasis and survival during IRI, and so induction of autophagy may promote the therapeutic effects of a potential treatment [34,35]. The balance is however difficult, as autophagy may both contribute to cell survival and induce cell death and exacerbate IRI in the kidney. Thus, the protective or detrimental character of autophagy depends on the ischemia duration and the phase of the IRI process [36].

Experimental investigations have demonstrated a beneficial effect of NAD^+^ boosting in various diseases [37–40]. The exact mechanisms by which such therapies are efficacious for remain, however, incompletely understood [41]. The NAD^+^ level is in cells adjusted by three biosynthetic pathways, the Preiss-Handler pathway (a process for utilizing dietary nicotinic acid), by de novo biosynthesis from tryptophan, and by the salvage pathway (a process that utilizes precursors, nicotinamide or nicotinamide riboside (NR), to generate NAD^+^) [40,42]. As such, it has been proposed that supplementation with NAD^+^ precursors could provide an effective strategy to improve patient outcomes in AKI [37,43,44].

AKI may either regenerate or convert to CKD [45–47]. Tubulo-interstitial fibrosis, a hallmark of CKD, is a progressive process, which is driven by activation of interstitial myofibroblasts and by an increase in pro-fibrotic and pro-inflammatory signals. Thus TGF-β1 signaling, reactivation of developmental pathways, and a decrease in the expression of protective factors, such as Klotho and bone morphogenetic protein 7 (BMP7) are found [8,48–55]. Klotho is an anti-aging protein, which is predominantly expressed in the kidney, where it functions as a co-receptor for FGF23, regulating phosphate and calcium reabsorption and calcitriol metabolism [56]. The soluble form of Klotho acts as a circulating factor with anti-inflammatory, antioxidant and anti-fibrotic properties [57]. Decreased renal expression of Klotho has been found in various models of AKI and CKD [50,58]. The anti-fibrotic effect of Klotho is mediated at least partially by a direct inhibitory effect on TGF-β1, Wnt and FGF2 signaling [59,60].

The aim of the present study was to examine the effects of NAD^+^ boosting by long-term administration of the NAD^+^ precursor, NR, on the renal pathophysiology involved in AKI. The study focused on a number of mitochondrial parameters, autophagy, expression of Klotho, and development of fibrosis in two experimental rat kidney IRI models. One where the kidney was examined 24 hours after IRI, or another where the kidney was examined 14 days after IRI, at a time when AKI had progressed to CKD.

## Results

### *Ngal*, a marker of AKI, and plasma biochemistry

Male Wistar rats were randomized to treatment with NR (a precursor of NAD^+^) or vehicle for 14 days. Then IRI was induced, and rats were sacrificed after 24 hours or 14 days. Another two groups of NR and vehicle given rats were sham operated and sacrificed 24 hours after the sham operation (for control).

**Fig 1:**
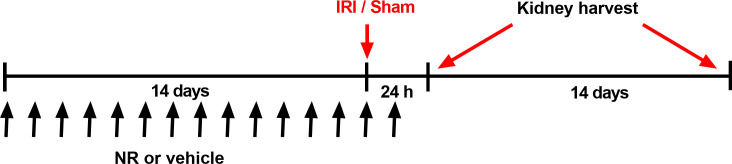
Experimental design of the model on prophylactic NAD^+^ boosting and bilateral renal ischemia-reperfusion injury (IRI). Animals were randomized to sham or IRI and given NR (500 mg/kg/d) or vehicle (water) from 14 days before the IRI or sham surgery to one day after surgery. Both renal arteries were clamped for 45 minutes. The rats were sacrificed after 24 hours or 14 days after IRI. NR: nicotinamide riboside.

The model is presented in Fig 1. To sum up, six groups were included in the study: Group 1: Sham + vehicle 24 h; Group 2: Sham + NR 24 h; Group 3: IRI + vehicle 24 h; Group 4: IRI + NR 24 h; Group 5: IRI + vehicle 14 days; and Group 6: IRI+ NR 14 days. NR and vehicle rats had similar basal weight in all groups (mean 341 ± 15 g), which increased slightly 14 days after surgery to (mean 368 ± 20 g).

**Fig 2:**
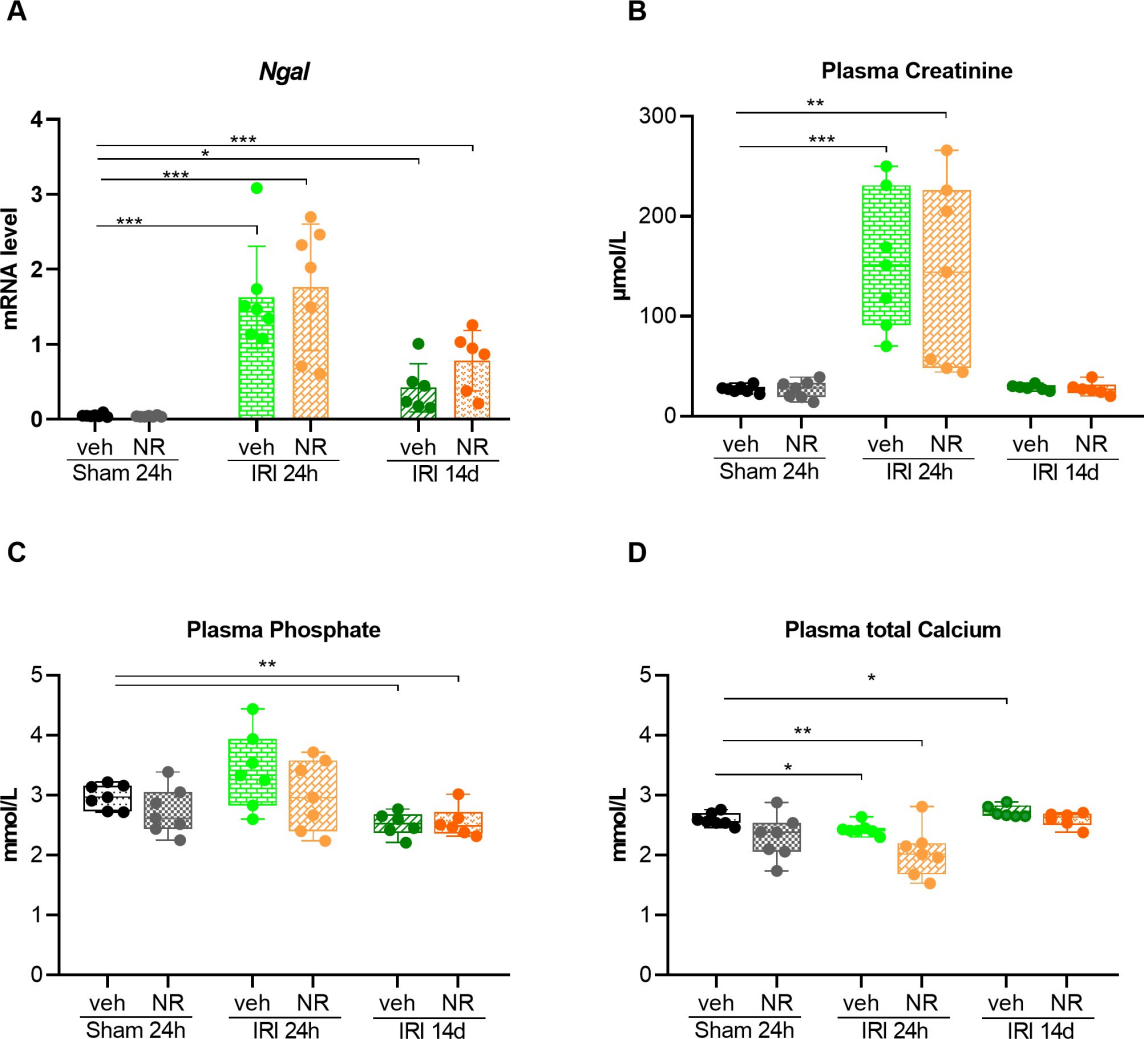
*Ngal*, an acute kidney injury marker and plasma biochemistry in sham animals and 24 hours and 14 days after IRI. Kidney expression of *Ngal* **(A)** and plasma biochemistry **(B, C, D)** are presented. *Ngal* expression was not affected by NR in the sham groups. The levels were similarly significantly increased after 24 hours after ischemia reperfusion injury (IRI) in the vehicle and sham groups and decreased to the same level 14 days after IRI. Plasma creatinine was similar in sham rats whether given NR or vehicle. IRI resulted in a significant increase in plasma creatinine after 24 hours with no difference between vehicle and NR groups.14 days after IRI, plasma creatinine became normal with no difference between the two groups. Plasma phosphate remained stable 24 hours after IRI at the level of sham rats in both vehicle and NR groups, and became significantly reduced 14 days after IRI. Basal plasma calcium was the same in vehicle and NR sham rats. Twenty-four hours after IRI significant hypocalcemia developed but increased again in the long term IRI groups. *Ngal* (neutrophil gelatinase-associated lipocalin). Mean ± SD; * p < 0.05, ** p < .001, *** p < 0.0001 (n = 7 in each group).

Kidney expression of *Ngal* (neutrophil gelatinase-associated lipocalin), a marker of acute kidney injury, and plasma biochemistry are presented in Fig 2. *Ngal* expression was not affected by NR in the sham groups. The levels were similarly significantly increased 24 hours after IRI (p<0.001) in the vehicle and sham groups and decreased to similar levels 14 days after IRI (p<0.01). Plasma creatinine was similar in sham rats whether given NR or vehicle. IRI resulted in a significant increase in plasma creatinine after 24 hours (p<0.01) with no difference between vehicle and NR groups. Fourteen days after IRI, plasma creatinine decreased to baseline values with no difference between the two groups. Plasma phosphate remained at the level of sham rats in both vehicle and NR groups 24 hours after IRI but was significantly reduced 14 days after IRI (p = 0.002 and p = 0.01). Basal plasma calcium was the same in vehicle and NR sham Twenty-four hours after IRI significant hypocalcemia developed (p = 0.01) but increased again in the long term IRI groups.

### Effect of NR administration on NAD^+^ boosting illustrated by NAD^+^ and NADH levels in the kidney

**Fig 3:**
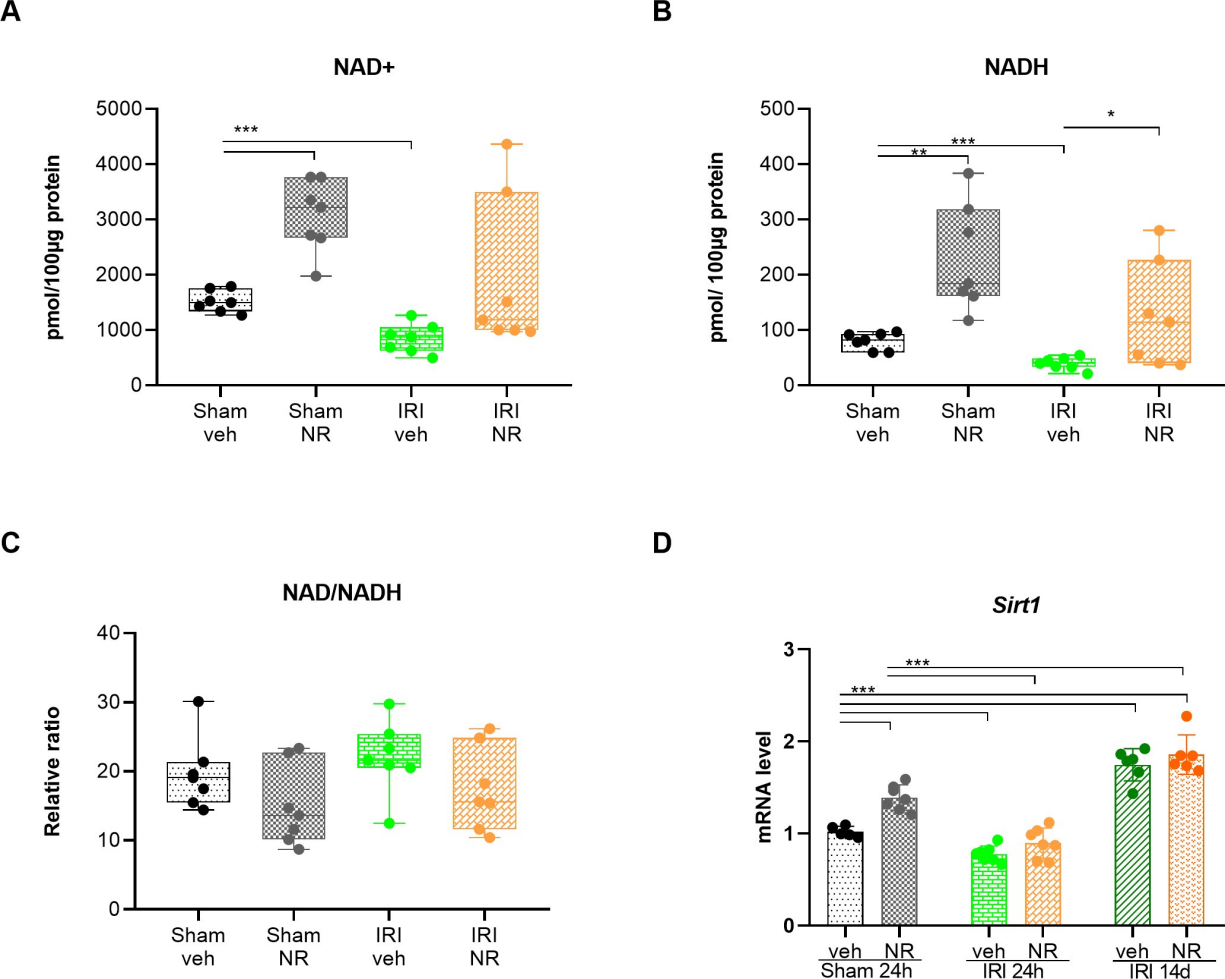
Effect of NR administration on NAD^+^ boosting. NAD^+^, NADH levels and Sirtuin1 expression in the kidney. NAD^+^ and NADH levels were measured in the kidney of sham+vehicle, sham+NR and IRI+vehicle and IRI+NR at 24 hours after IRI or sham. (**A-B**) NAD^+^ and NADH levels were significantly elevated in NR groups compared with the sham groups. IRI+vehicle rats had a pronounced significant reduction in the NAD^+^ and NADH levels compared with the sham+vehicle group. Also, NADH levels were significantly decreased in the IRI+vehicle as compared with IRI+NR group. NR rescued the fall in NAD^+^ and NADH levels after IRI. (**C**) The NAD/NADH ratio was unchanged in all the groups. **(D)** The expression of *Sirt1* was significantly increased in sham+NR rats. IRI resulted in a significant fall in *Sirt1* levels to a similar level. Mean ± SD; * p < 0.05, ** p < .001, *** p < 0.0001 (n = 7 in each group).

In normal physiological conditions, NAD is cycled in a redox couple NAD^+^/NADH. NAD^+^ is reduced to NADH. Conversely, NADH oxidation occurs in the first step of the mitochondrial oxidative phosphorylation mediated by complex I, converting NADH to NAD^+^ [37]. Administration of the NAD^+^ precursor NR for 14 days significantly boosted kidney NAD^+^ levels (p<0.001), as compared to the sham+ vehicle group (Fig 3A). NADH became significantly increased as well (p = 0.002), and the ratio NAD^+^/NADH was similar between the sham+vehicle and the sham+NR rats (Fig 3B and 3C). This indicates that treatment with NR effectively increased kidney NAD^+^ while maintaining the metabolic balance between NAD^+^ and NADH. Twenty-four hours after IRI, kidney NAD^+^ levels were significantly decreased in vehicle animals (p<0.001), however, the levels of NR treated rats were similar to the basal levels of NAD^+^ in the kidneys of sham+vehicle rats. The NAD^+^/NADH ratio was maintained. These results indicate that NAD^+^ in the kidney tissue was significantly increased by treatment with NR. Also, NAD+ reduction induced by IRI was ameliorated.

### Effect of NAD^+^ boosting on Sirtuin 1

Sirtuins are a family of NAD^+^-dependent histone deacetylases involved in the regulation of metabolism, inflammation, DNA damage and repair, oxidative stress, and mitochondrial energy homeostasis. SIRT1 has been shown to exert protective effects in kidney diseases and in AKI, mainly due to its activity on mitochondrial function and to its activation of *pgc-1α*. Furthermore, sirtuins have been shown to have anti-fibrotic functions and to ameliorate the progression of AKI to tubule-interstitial fibrosis [61]. The expression levels in the kidney of *Sirt1* are presented in Fig 3D.

Treatment with NR resulted in a significant increase (p<0.001) in the expression of *Sirt1* in the kidney of sham rats. *Sirt1* expression decreased significantly in the IRI+vehicle 24 h (p<0.001). In the IRI+NR 24 h group the expression of *Sirt1* decreased as well (p<0.001), although only to a level similar to the baseline of the sham+vehicle rat kidney, which was significantly lower than the one of sham+NR 24 h rats (p<0.001). In both the IRI+vehicle 14 d and IRI+NR 14 d groups, the expression levels of kidney *Sirt1* were significantly increased (p<0.001).

### Effect of NAD^+^ boosting on autophagy

Autophagy is a lysosomal dependent catabolic process that begins by encapsulating the damaged organelle or protein in autophagosomes. Next, it can fuse to a lysosome for degradation. LC3B and P62 are two of the most examined biomarkers for autophagy. In the case of oxidative stress or hypoxia, the free form of lipidation of microtubule-associated protein-1 (LC3B-1) binds in the cytoplasm to phosphatidylethanolamine to form LC3B-II, which is localized on the phagocytic membrane [36]. LC3B-II is degraded in the lysosomes. Therefore, the amount of LC3B-II at a specific time is dependent on both autophagosome production and degradation rate. This continuous autophagic flux affects another biomarker, which is important for the resulting autophagy, the autophagy degradation substrate, P62 [62]. The amounts of LC3B-II and P62 reflect the autophagic activity.

**Fig 4:**
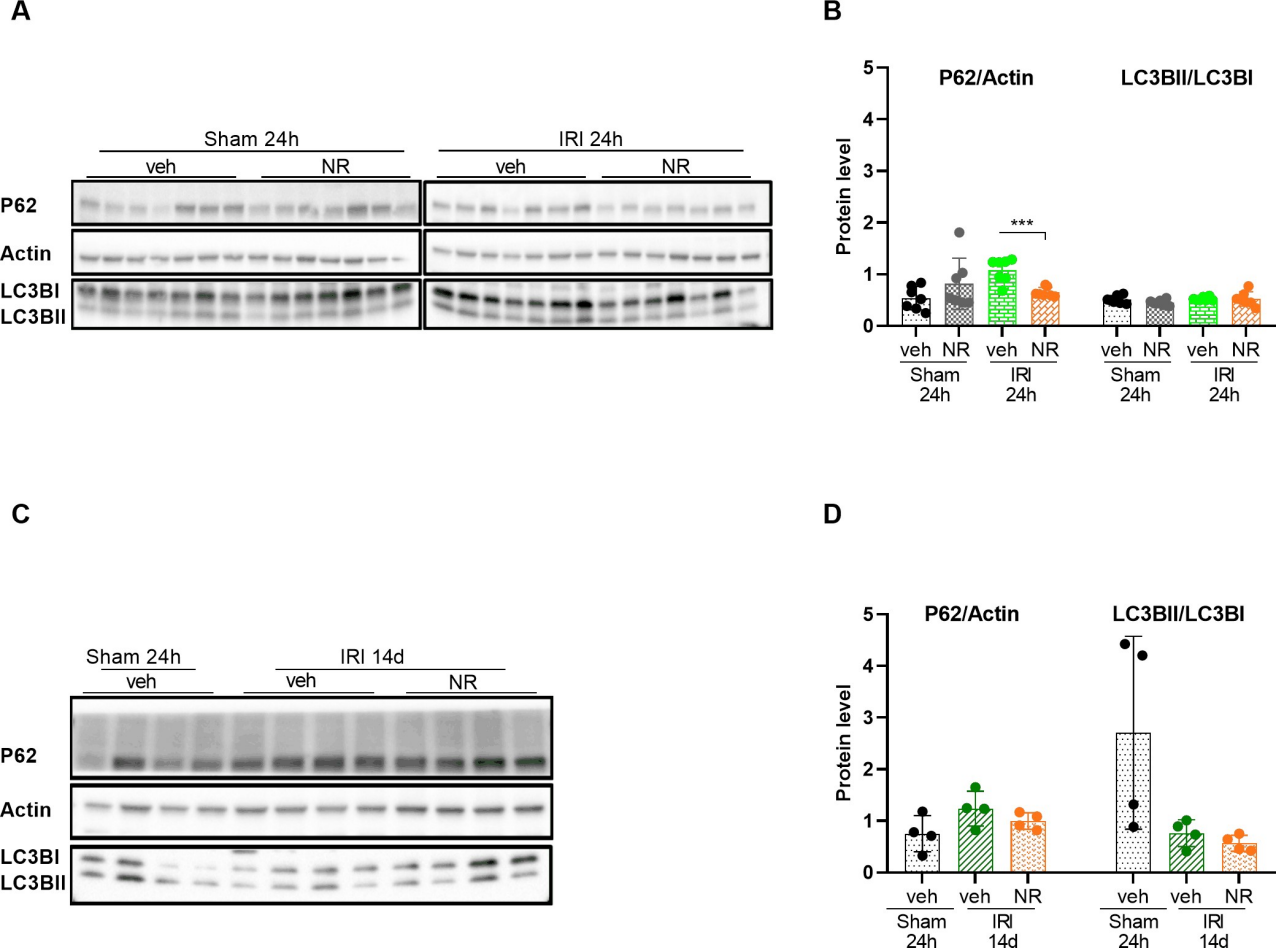
Effect of NAD^+^ boosting by NR on autophagy in the kidney. Western blot analysis and quantification of P62 protein, a marker for autophagy, showed that NAD^+^ boosting in sham group did not interfere with the levels of P62. A significant increase in P62 level was found in the IRI+vehicle compared to the IRI+NR group after 24 hours (**A** and **B**). The ratio between LC3BII/LC3BI was unchanged in all groups. Western blot analysis and quantification of P62 levels and LC3BII/I ratio 14 days after IRI are presented in (**C** and **D**). The level of P62 became normalized in the IRI groups. The ratio of LC3BII/ LC3BI remained unchanged. Mean ± SD; * p < 0.05, ** p < .001, *** p < 0.0001.

The protein level of P62 and the ratio of LC3B-II/LC3B-I were examined in the kidney tissue, and the results are presented in Fig 4. P62 was significantly increased in the IRI+vehicle 24 h rats compared to sham+vehicle rats, this was confirmed in an additional investigation where the samples were run on the same gel (S1 Fig). The increased P62 level was rescued by treatment with NR as it was significantly lower in the IRI+NR 24 h rats (p<0.001). NAD^+^ boosting did not have an impact on the ratio of LC3B-II/LC3B-I in the two groups of sham rats and in the two IRI 24 h groups. P62 level recovered to baseline values in both IRI+vehicle 14 d and IRI+NR 14 d rats, and the ratio LC3B-II/LC3B-I was maintained normal. These results indicate that autophagy is altered 24 h after IRI with inhibition of autophagosome degradation and further show that NR treatment might ameliorate this pathology. Normal levels of autophagy were re-established 14 days after IRI.

### Mitochondrial dysfunction after IRI, effect of NAD^+^ boosting by NR

**Fig 5:**
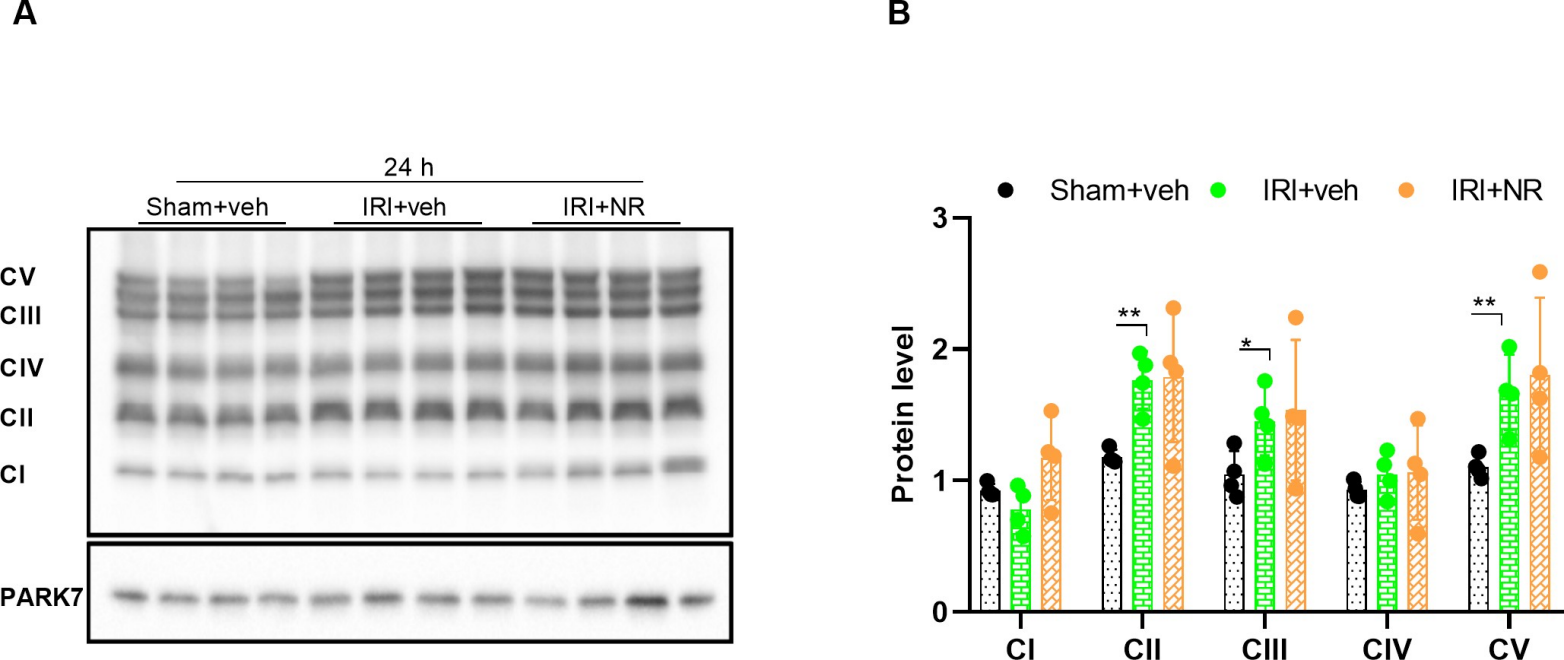
Effect of NAD^+^ boosting by NR on the mitochondrial oxidative phosphorylation (OXPHOS) complexes. Western blot **(A)** and quantifications (**B**) of the subunits of the mitochondrial OXPHOS complex I-V demonstrated that subunits II, III, and V were significantly increased by IRI but not further affected by NAD^+^ boosting. The levels of OXPHOS complex I-V were normalized to the housekeeping protein, PARK7. Mean ± SD; * p < 0.05, ** p < .001, *** p < 0.0001.

The oxidative phosphorylation system (OXPHOS) in the inner mitochondrial membrane is an intricate process that consists of five multi-peptide complexes (CI-CV) composed of many different structural proteins, which are encoded by nuclear and mtDNA. They require assembly protein factors for proper function. Changes in the protein level of OXPHOS subunits may reflect an altered function of the electron transport chain. The abundance of selected OXPHOS subunits was examined. The mitochondrial OXPHOS complex II, III, V (Fig 5) were significantly more abundant in the IRI+vehicle 24 h and IRI+NR 24 h group (p = 0.002, p = 0.04 and p = 0.009) than in the sham+vehicle group. Elevated complex II is an indication of an increased ROS production, which supports the observation that severe tubular injury was observed 24 hours after IRI. Taken together, these results demonstrated that IRI 24 hours later leads to upregulation of the OXPHOS protein subunits, probably as a compensatory transcriptional response to mitochondrial dysfunction [63,64].

### NAD^+^ boosting, effect on kidney tubular markers

**Fig 6:**
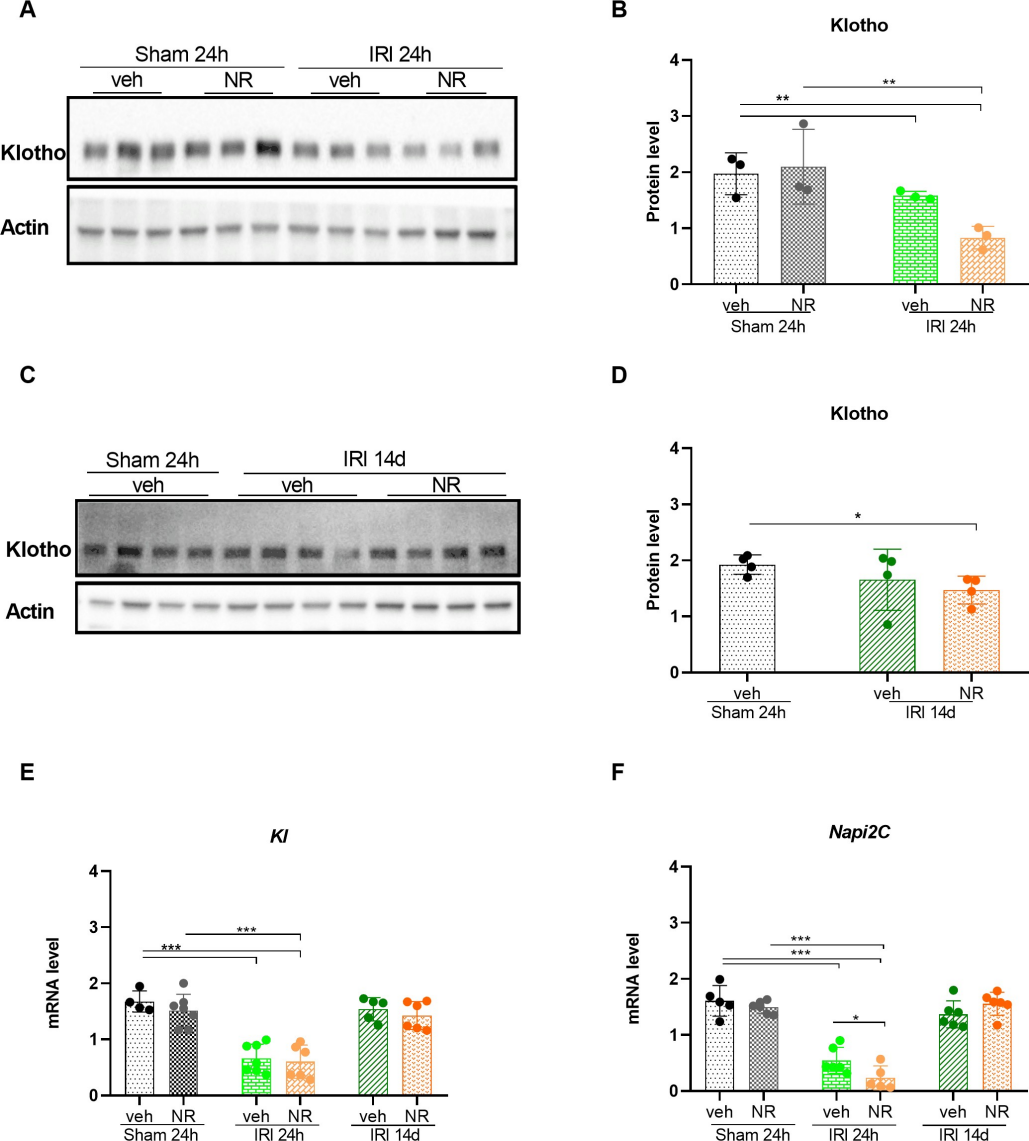
Effect of NAD^+^ boosting by NR on *Klotho* and *Napi2c* in the kidney. Western blot of klotho protein in the kidney of sham+vehicle, sham+NR and IRI+vehicle and IRI+NR at 24 hours **(A)** and its quantification **(B)** normalized to actin. NAD^+^ boosting did not affect klotho levels in the sham groups. Klotho became significantly decreased by IRI in both the vehicle and NR groups after 24 hours. **(C)** Western blot of klotho in the kidney of IRI+vehicle and IRI+NR after 14 days and its quantification **(D)** showed that klotho in the IRI+NR 14 days group was significantly reduced as compared with the sham group. **(E)** The mRNA levels of *Kl* were similarly reduced at the protein level. **(F)** mRNA of *Napi2c* was significantly decreased 24 hours after IRI in both groups and administration with NR resulted in a further decrease. *Napi2c*: sodium phosphate cotransporter 2. Mean ± SD; * p < 0.05, ** p < .001, *** p < 0.0001.

Klotho is predominantly expressed in the distal tubules in the kidney and a small amount in the proximal tubules [65–67]. Decreased expression of klotho is associated with various kidney injuries, while administration with recombinant klotho protein or klotho overexpression protects against the injury [58,68]. Therefore, the effect of NAD^+^ boosting on the kidney klotho levels was examined in the IRI model, and the expression levels of klotho protein and mRNA are shown in Fig 6. Boosting of NAD^+^ by NR did, however, not change the expression of klotho in sham animals (Fig 6). Klotho protein and mRNA levels were significantly decreased in the IRI+vehicle 24 h group (protein: p = 0.003; mRNA: p<0.001), and NAD^+^ boosting with NR did not rescue this fall in klotho in the IRI+NR 24 h group. Fourteen days after IRI, the expression of the klotho gene was returned to normal levels in both the IRI+NR 14 d and IRI+vehicle 14 d groups, but the protein level still was slightly reduced (p = 0.02) in the IRI+NR 14 d group (Fig 6).

Proximal tubular segments are most prone to injury after AKI [69]. *Napi2c* is exclusively expressed in fully differentiated proximal tubular epithelial cells [70]. The level of *Napi2c* was significantly downregulated in both the IRI+NR 24 h and the IRI+vehicle 24 h groups (p<0.001), with a significantly lower level in the IRI+NR 24 h than in the IRI+vehicle 24 h group (p = 0.05). The expression of *Napi2c* was not affected by NAD^+^ boosting in sham rats (Fig 6). The expression levels of *Napi2c* mRNA became normal after 14 days after IRI in both vehicle and NR animals. These results indicate that the expression of tubular markers was significantly reduced 24 h after IRI and NAD^+^ boosting with NR did not rescue this event. On the contrary, the expression levels of the tubular markers were further diminished in NR treated rats (p = 0.05). Furthermore, the expression of klotho, the kidney protective protein, was still reduced 14 days after IRI, indicating the presence of persistent damage of the kidney tissue in this model despite NAD^+^ boosting.

### NR boosting and IRI, effects on the de novo NAD^+^ biosynthesis pathway

Quinolinate phosphoribosyltransferase (QPRT) is a key enzyme in the NAD+ de novo pathway for converting quinolinic acid to NAD^+^ [43]. *Pgc-1α* is a master regulator of mitochondrial biogenesis and downstream targets include nuclear-encoded electron transport chain protein cytochrome C4 (*Cox4*) and antioxidants such as *SOD2* and Cat [71].

**Fig 7:**
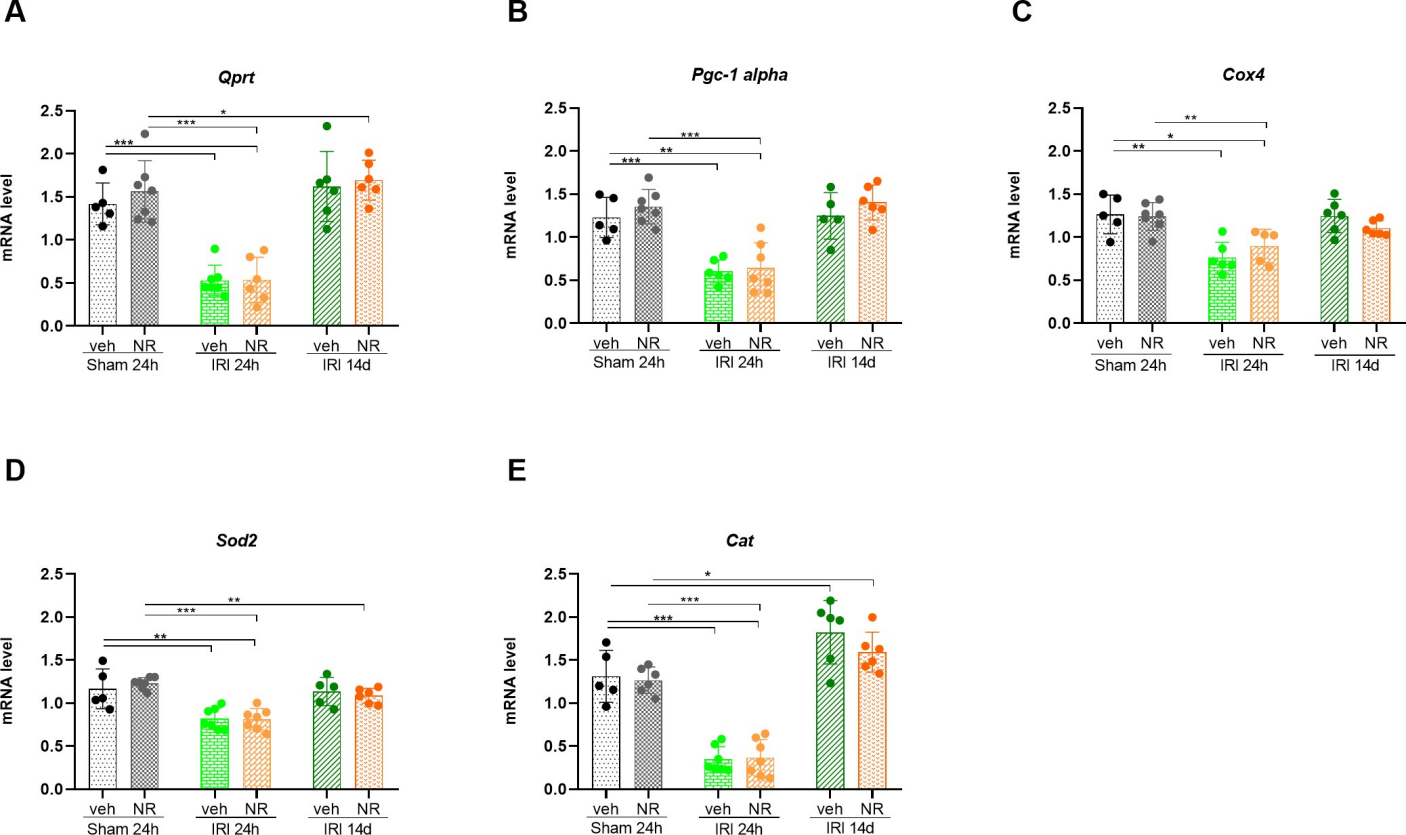
Effect of NAD^+^ boosting by NR on genes related to the de novo NAD^+^ pathway in the kidney. The expression levels of genes related to the de novo biosynthesis pathway of NAD^+^ were examined. *QPRT* is a key enzyme in the NAD^+^ de novo pathway for converting Quinolinic acid to NAD^+^. It was significantly downregulated by IRI to the same extent in both the IRI+Vehicle and IRI+NR 24 hour groups. The levels became normalized or upregulated 14 days after IRI **(A)**. The expression level of *pgc-1α* was also significantly downregulated after 24 hours in the IRI+vehicle and IRI+NR groups, as compared to the sham groups **(B)**. **(C)** *Cox4* was significantly downregulated in both IRI groups after 24 hours compared to the sham groups and became normal after 14 days **(C)**. The two antioxidants *Sod2* and *Cat*, downstream for *pgc-1α*, were significantly downregulated in both IRI groups after 24 hours compared with the sham groups. Finally, were also levels of *Sod2* downregulated in the IRI+NR-14 days group compared to sham+NR **(D and E)**. *Qprt*: Quinolinate Phosphoribosyltransferase; *Pgc-1α*: Peroxisome proliferator-activated receptor gamma coactivator 1α; *Cox4*: Cytochrome c oxidase subunit 4; *Sod2*: Superoxide dismutase 2; *Cat*: Catalase. Mean ± SD; * p < 0.05, ** p < .001, *** p < 0.0001.

The expression levels of these genes are shown in Fig 7. There was no difference in the expression levels of *Qprt* and *pgc-1α* between sham NR and vehicle animals. 24 hours after IRI both *Qprt* and *pgc-1α* were significantly downregulated (p<0.001). Fourteen days after IRI the expression levels of *pgc-1α* and *Qprt* were recovered in both IRI+NR and IRI+vehicle rats, although NR rats had slightly, but not significantly higher *Qprt* levels (p = 0.085). NR had no effect on *Cox4* expression levels in the sham rats. The expression of *Cox4* was significantly downregulated 24 h after IRI (p = 0.002) and found to be normal 14 days after IRI in both NR and vehicle rats. The downstream antioxidant enzymes for *pgc-1α*, *Sod2*, and *Cat* were not affected by NAD^+^ boosting by NR in sham rats. Twenty-four hours after IRI the expression of *Sod2* and *Cat* decreased significantly in the NR and vehicle groups, compared with sham animals (*Sod2*: p = 0.01; *Cat*: p<0.001). Fourteen days after IRI *Sod2* and *Cat* expression levels became normalized in both groups compared with sham+vehicle animals. These results indicate that the de novo NAD^+^ biosynthesis pathway and the related mitochondrial genes are impaired in AKI. Boosting of NAD^+^ in the kidney tissue did, however, not affect the de novo NAD^+^ biosynthesis pathway related genes.

### NAD^+^ boosting and expression of factors related to IRI induced kidney fibrosis

**Fig 8:**
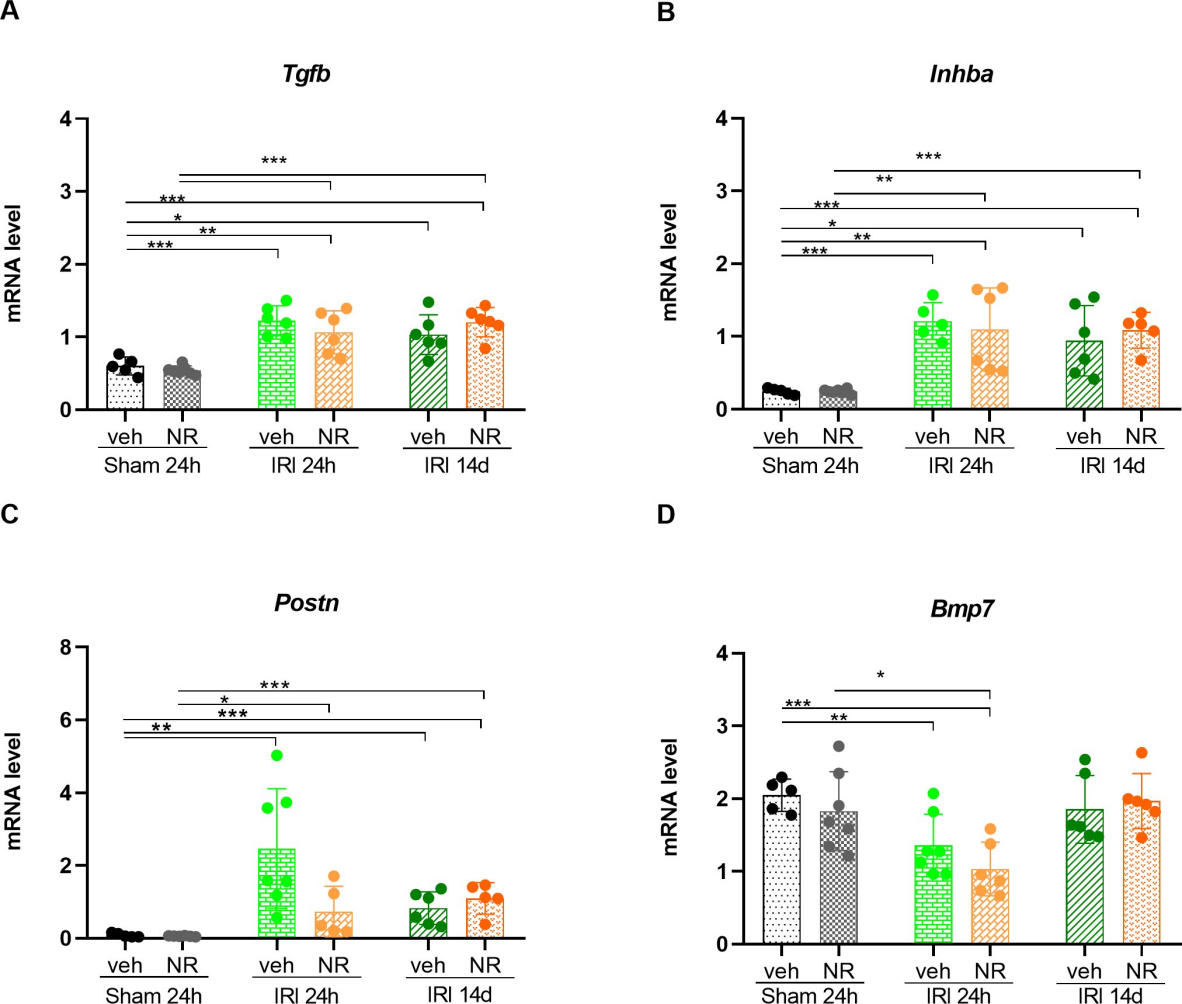
NAD^+^ boosting by NR and expression of fibrogenic markers in the kidney. The four figures depict the expression level of *Tgfb1*, *Inhba*, *Bmp7*, and *Postn*. The relative mRNA level in those genes was not changed by NR administration in sham groups. Significant upregulation to the similar level of the expression of *Tgfb1*, *Inhba* and *Postn* was seen after 24 hours in both IRI groups compared with sham groups. *Tgfb1*, *Inhba* and *Postn* were still upregulated after 14 days post-IRI in both groups compared with sham groups **(A—C)**. The level of *Bmp7* was significantly downregulated at 24 hours post-IRI in both groups compared with sham groups. The *Bmp7* level was normalized after 14 days post-IRI compared with sham **(D)**. *Tgfb*1: Transforming growth factor-beta1; *Inhba*: Inhibin subunit beta A; *Postn*: Periostin; Bmp7: Bone morphogenetic protein 7. Mean ± SD; * p < 0.05, ** p < .001, *** p < 0.0001.

**Fig 9:**
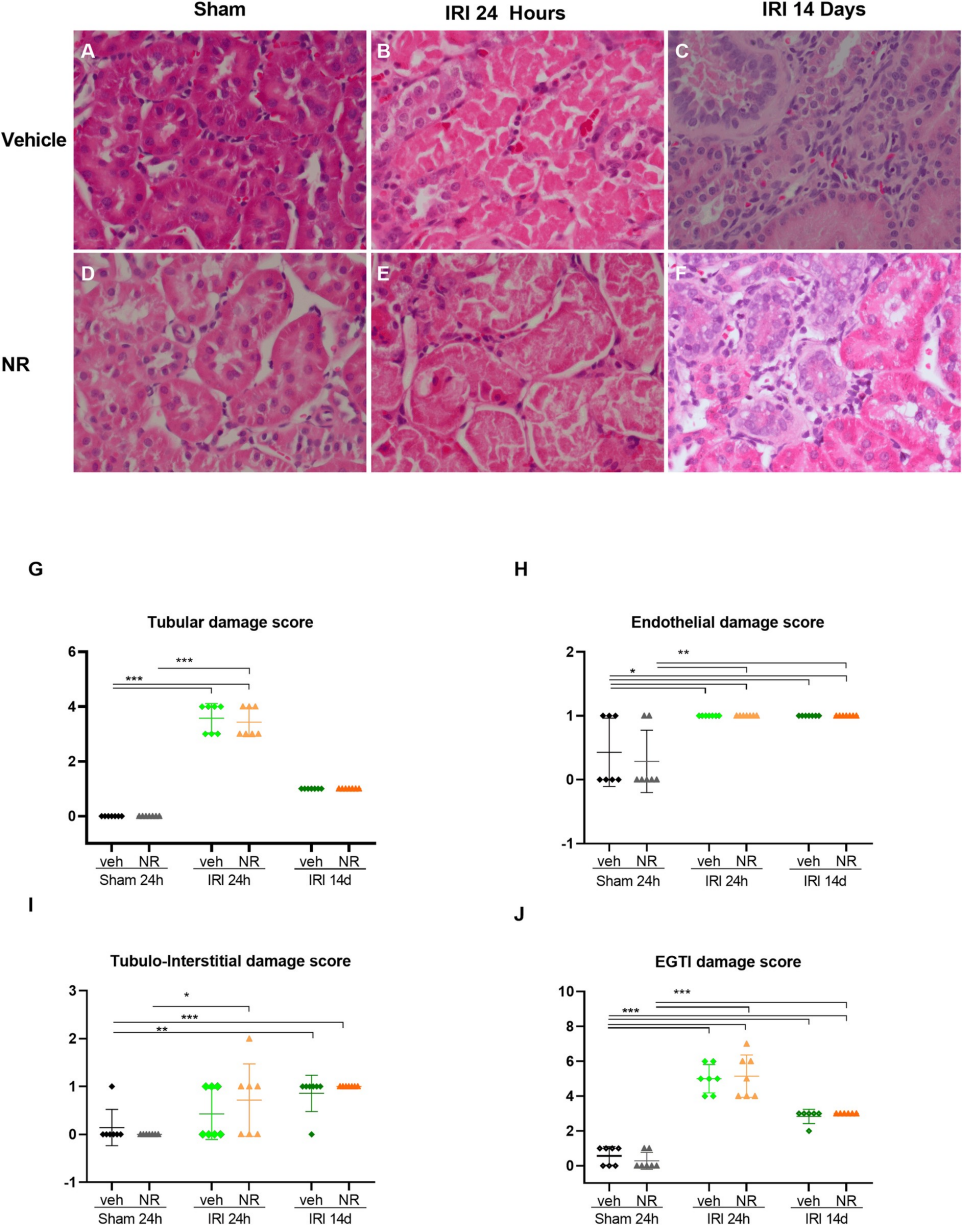
Effect of boosting NAD^+^ by NR on the kidney histological appearance in IRI. Representative kidney cortex sections from sham rats, and IRI rats after 24 hours and 14 days treated with NR or vehicle, H&E (400) **(A-F).** Significant damage of the kidney cortex was seen in both IRI groups after 24 hours with inflammation, cast-formation, and severe appearance of necrosis in the tubular cells, mild endothelial swelling and tubular/interstitial inflammation, and hemorrhage in the tissue. The glomeruli were not significantly affected. Fourteen days after IRI the tubular changes were less severe, but development of interstitial fibrosis appeared. The histological changes were scored with focus on tubular, glomerular, endothelial and tubulo-interstitial damages 24 hours and 14 days after IRI in NR and sham rats, demonstrating that even though severe changes were induced, there was no significant improvement of boosting NAD^+^ by NR **(G-J)**. EGTI damage score: Endothelial, Glomerular, Tubular, and Interstitial damage score. Mean ± SD; *p <0.05, **p <0.001, ***p <0.0001.

TGF-β1 is a major contributor to the induction of renal fibrogenesis, exerting its action via apoptosis of tubular cells, induction of epithelial-to-mesenchymal transition (EMT), and decreased degradation of extracellular matrix [72]. Activin A is a homodimer of two inhibin-β subunits and is another member of *Tgf-β* superfamily involved in renal development and repair [50,73–75]. The renal expression of activin A disappears in the adult kidney, but it is induced in acute renal injury and progressive renal fibrosis [50,76]. Periostin is another factor participating in fibrogenesis and EMT [77]. The effect of NR on the expression of the fibrogenic markers, *Tgf-β1*, *Inhba* and *Periostin* was examined (Fig 8). No difference was observed in the basal expression level of these genes in the sham +NR and vehicle rats. The expression of *Tgf-β1* was significantly upregulated (p = 0.01 and p<0.001) and the expressions of *Inhba* and *Periostin* were significantly induced in the IRI 24 h groups with no significant differences between NR and vehicle rats (Fig 9A). In the IRI 14 d groups, these fibrogenic markers remained significantly upregulated (NR: p<0.01 and vehicle: p = 0.01), but no difference was found in the kidneys of the two groups.

BMP7 is a developmental and differentiation factor in the kidney [78]. In the adult kidney expression of BMP7 is retained and BMP7 is considered essential for maintaining tubular epithelial integrity [8,79]. In various kidney diseases tubular epithelial expression of BMP7 is decreased and treatment with exogenous BMP7 stimulates kidney repair and prevents progression of kidney disease [78]. The effect of boosting with NAD^+^ on the expression of kidney *Bmp7* was investigated (Fig 8) in the present study. No difference was observed in the basal expression of *Bmp7* between the sham+ NR and the sham+vehicle groups. In the IRI+NR and IRI+vehicle 24 h groups *Bmp7* was significantly downregulated (p<0.001 and p = 0.008) with no difference between the two groups. The expression of *Bmp7* was reestablished in both IRI+NR 14 d and IRI+vehicle 14 d groups.

The expression of the anti-inflammatory macrophage marker, *CD206* was increased in the NR group at 24 post IRI (S2 Fig), while expression of the inflammation marker *CCR2* was severely increased in the kidney 24 hours post IRI and still increased after 14 days with no effect of NR. The expression of the pro-inflammatory markers, *CD38* and cytokine *IL6* in the kidney tissue became increased at 24 hours and 14 days post IRI. These results indicate together with induction of activin A (Fig 8), that the classically activated macrophage M1 pathway is pre-dominant in IRI and independent of NAD+ boosting.

Thus our results show that the kidney IRI already after 24 hours caused a pro-fibrotic shift in the pattern of expression of kidney genes and that the expression levels of the pro-fibrotic genes (*Tgf-β1*, *Inhba* and *Periostin)* 14 days after IRI were still significantly elevated in both NR and vehicle rats, indicating that NAD^+^ boosting did not protect against transition from AKI to chronic kidney fibrosis in IRI rat model.

### NAD^+^ boosting and IRI, effect on kidney histology

Kidney IRI causes pronounced histological damage in kidney tissues. To examine the potential protective effect of NAD^+^ boosting by NR at the histological level, kidney slices were stained with H&E, PAS, and Trichrome in the IRI and sham groups. A representative H&E staining for each group is shown in Fig 9. The cellular components, endothelial, glomerular, tubular, and interstitial, were scored by a comprehensive histological scoring system (EGTI) [80]. The quantification for each component and the overall scoring is shown in Fig 9. Vehicle and NR sham rats were all histologically normal. The kidneys from IRI+vehicle and IRI+NR 24 h showed a high degree of damage, primarily in the renal cortex, with tubular necrosis, focal dilatation, and cast formation (Fig 9). These damages were mainly located in the proximal tubules but also seen in distal tubules (Tubular score 5). Endothelial hypertrophy was present and only a mild degree of damage in other components of the nephrons was observed (Interstitial score 1).

The kidneys from IRI+NR or vehicle 14 d were characterized by a mild degree of chronic changes that were not different between the two groups. Thus, showing generally mild chronic tubular-interstitial damages (Interstitial score 1) with focal tubular atrophy (Tubular score 1), slight thickening of the tubular basement membranes, and mild interstitial fibrosis and focal inflammation with mononuclear cells (Fig 9). The glomeruli were not affected by any interventions (Glomerular score 0). These results indicate that prophylactic treatment with NR did not significantly affect histological appearance at 24 h after IRI. Additionally, the IRI+NR 14 d group developed indications of fibrosis, and as such, did prophylactic NR not have a specific effect on the histological appearance in the kidney.

## Discussion

The present model of acute kidney ischemia-reperfusion injury (IRI) is a model for AKI that resulted in a rapid decrease in kidney function with a significant increase in plasma urea and creatinine as well as in disturbed ion homeostasis leading to abnormal calcium levels after 24 hours. The functional abnormality was associated with decreased expression of tubular markers *klotho*, *Napi2c*, and *BMP7*, disturbed mitochondrial biogenesis and autophagy, and to a pro-fibrotic shift in the pattern of expression of the kidney genes *Tgfb1*, *Inhba* and *Periostin*. Histological examination revealed severe endothelial and tubular damage, necrosis, and inflammation. After 14 days, signs of transition to progressive renal fibrosis were seen with a persistent increase in the expression of pro-fibrotic genes, tubular atrophy, interstitial fibrosis, and focal inflammation.

A large body of literature has demonstrated the importance of the intracellular biosynthetic pathways, related to NAD^+^ in the amelioration of kidney injury. AKI was found associated with decreased synthesis and levels of NAD^+^, which might contribute to the pathogenesis of ischemic kidney disease [44]. We therefore hypothesized that boosting kidney NAD^+^ by long term administration of NR might have a kidney protective effect in AKI and might prevent the transition from AKI to CKD in experimental IRI. The relatively high dose of NR used in the present investigation was chosen according to previous studies [81,82] showing an effect of a similar NAD+ boosting regime on cerebellum and liver in *in vivo* models.

NR is a soluble and orally bioavailable endogenous molecule, making it the molecule of choice for animal experiments and human clinical trials. Furthermore, NR has been shown to be more efficient in boosting NAD^+^ than other NAD^+^ precursors such as NAM [83,84], possibly due to increased intestinal uptake [85,86]. In order to ensure that all animals got the same amount of the NR solution, we gave the drug by oral gavage fourteen days before the surgery in order to boost the NAD^+^ pool. In the present study, experimental AKI was induced by IRI, which rapidly led to a decrease in kidney NAD^+^ levels, in line with previous investigations that found decreased NAD^+^ in the acute phase of ischemia [43,44]. Kidney NAD^+^ was considerably augmented by long term administration of the NAD^+^ precursor, NR, and the fall in NAD^+^ after IRI was ameliorated. Administration of NR not only efficiently boosted kidney NAD^+^ but also enhanced the expression of *Sirt1* and mitochondrial transcription factor A (*Tfam*) mRNA levels (S3 Fig). The upregulation of *Sirt1* might potentially be beneficial for the kidney. SIRT1 has previously been shown to be widely expressed in the kidney, in tubular cells and podocytes, regulating mitochondrial biogenesis, TGF-β, and apoptosis signaling [31,61,87]. SIRT1 might be involved in sodium and water handling and in blood pressure control [61,88]. Enhanced *Sirt1* expression might potentially have a positive effect in AKI by maintaining mitochondrial function, apoptosis and by decreasing the epithelial sodium reabsorption via repressing the production of the epithelial sodium channel [61]. TFAM is a key activator of mitochondrial transcription and DNA replication. These potentially positive effects of NR on *Sirt1* and *Tfam* expressions need to be looked for in a different, less devastating model of kidney injury than the present IRI model. In this respect, it should be noted that no harmful effect of long-term administration of NR was observed on the kidney in the present investigation.

The mRNA expression of NAD+ consuming enzymes *Sirt1* and *CD38* was disturbed both 24 hours and 14 days after IRI, however with no difference between NR and vehicle, indicating that AKI has a significant effect on the expression of the NAD+ consuming enzymes both in the short and long term. This effect is however, not changed by NAD+ boosting.

The rescued elevated levels of NAD^+^ and *Sirt1* found at 24 hours after IRI did not have a protective effect on the kidney function or on indicators of the injury and fibrosis in the kidney IRI model. This is in contrast to other studies in mice with shorter ischemia duration where NAD+ precursor treatment ameliorated AKI [89,90]. Thus, the effect of NAD+ boosting is model dependent. However, in agreement with the results of the present investigation, previous studies, although demonstrating an effect of NAD+ on AKI parameters, NAD+ boosting did not prevent progression of CKD.

The de novo NAD^+^ biosynthetic pathway has previously been considered as an important minor contributor to the NAD^+^ synthesis. Recent studies, however, indicate a potential role of the de novo pathway in kidney embryogenesis, as rare loss-of-function mutations in the de novo enzymes 3-hydroxyanthranilate-3,4-dioxygenase (HAAO) or kynureninase (KYNU) were associated with major kidney anomalies [91]. QPRT has been proposed as a mediator of kidney stress. Impairment of the de novo NAD^+^ biosynthetic pathway, expressed by a decrease in QPRT has been shown in IRI. Genetic deletion of this enzyme was associated with increased susceptibility to ischemia [43]. In the present study, the de novo pathway was shown to be affected as the levels of *Qprt* were significantly reduced 24 hours after IRI, and this defect was not rescued by boosting NAD^+^ levels in the kidney by NR.

Mitochondrial biogenesis is another essential factor for the recovery of the injured kidney, and pathways associated with stabilizing the homeostasis of the mitochondrial biogenesis might improve AKI [92]. *Pgc-1α* does potentially attenuate the kidney injury by promoting mitochondrial respiration and increasing the recovery of genes related to the mitochondrial electron transport chain. *Pgc-1α* is the primary regulator of mitochondrial biogenesis. It activates transcription factors, such as Nrf1, NRF2, ERR-α, and PPAR-α, which mediate the transcription of mitochondrial DNA, antioxidants, and genes involved in the biogenesis [93]. The NAD^+^ precursors, resveratrol, and other SIRT1 activators, such as NR, has been shown to stimulate the expression and activation of *pgc-1α* through *the Sirt1* deacetylation pathway [94–98]. Activation of *pgc-1α* leads to reduced oxidative damage and cell death and accelerates the recovery of the kidney mitochondria and tubular homeostasis. Two weeks of NR administration had however, no effect on the levels of *pgc-1α* or on its downstream targets in the present model of IRI. This might be because AKI results in an upregulation of kidney di-/tri-acylglycerols [99] due to reduced de novo NAD^+^ biosynthesis, which causes a significant reduction in *pgc-1α*. Thus, the amount of rescued *Sirt1* in the kidney may not have been sufficient to increase *pgc-1α*, which regulates the expression of the de novo NAD^+^ biosynthesis gene, *Qprt*. As such, our results indicate that the de novo pathway in the AKI model used in the present investigation was disrupted and that the rescued NAD^+^ after 24 hours, therefore, did not affect *pgc-1α* or its downstream target genes. The results of the present investigation further indicate that the de novo pathway is a key pathway in the kidney and that a very high increase in SIRT1 might be required in order to stimulate *pgc-1α* enough to affect the regulation of the de novo pathway.

Autophagy is another important pathway which is involved in AKI [100]. This is due to it’s cytoprotective effect in response to stress factors. Kidney-specific deletion of autophagy genes in the proximal tubules resulted in tubular injury [101]. NAD^+^ is involved in autophagy, and reduced NAD^+^ potentially regulates the degree of autophagy in kidney tissues [33,102,103]. In the present investigation, NR administration reduced the increased levels of P62 that were observed 24 hours after IRI. This might ameliorate the degree of autophagy [104], as NAD^+^ boosting by NR increased the autophagic flux and thereby reduced the accumulation of autophagosomic P62. Interestingly, autophagy may however also have the opposite effect, depending on the severity of the ischemic injury. Thus, extended ischemic periods may increase the likelihood of tipping the balance toward autophagy-dependent cell death. The duration time of ischemia was 45 min in the present IRI model. While *in vivo* models autophagy may have a protective effect, when the ischemic duration is limited to 25–40 minutes, but if ischemia is prolonged to 40–60 minutes, a destructive effect has been observed [36]. This could be the case in the present model.

The mitochondrial response to NAD^+^ was studied by the OXPHOS complex II, III, V that were significantly more abundant in the IRI+vehicle group after 24 hours. Administration of NR provided similar, although no significant changes. Thus, we speculate that IRI 24 hours later leads to upregulation of the OXPHOS protein subunits, probably as a compensatory transcriptional response to mitochondrial dysfunction.

IRI, which in the present investigation was used as a model for acute kidney injury, resulted in a significant reduction of the potential kidney protective factors, klotho and BMP7. Strategies that are enhancing or maintaining the biosynthesis of klotho and BMP7 in kidneys, which are exposed to injury, will be of great importance, but are still lacking. The present results did however clearly show that NAD^+^ boosting did not preserve the expression of klotho or BMP7.

### In conclusion

Prophylactic NR for 14 days prior to IRI significantly boosted kidney NAD^+^ levels as well as *Sirt1* and ameliorated the fall in NAD^+^ detected after IRI. Prophylactic NAD^+^ boosting by NR significantly reduced the levels of P62 and had a significant impact on the degree of autophagy in the kidney. IRI resulted in AKI with tubular damage, decreased expression of klotho and BMP7, induction of pro-fibrotic markers, *Tgfb1*, *periostin*, and *Inhba*, mitochondrial dysfunction, reduced kidney function, and later in the transition to chronic kidney fibrosis, which was not ameliorated by NR.

## Methods

### Animals

Two-month-old male Wistar rats (Taconic A/S, Ejby, Denmark) were housed in a temperature-controlled environment with a 12-hour light-dark cycle. These animals had free access to water and standard rodent chow containing 0.9% Calcium, 0.7% Phosphate, and 600 IU vitamin D3 per kg food (Altromin Spezialfutter, Germany) were used.

### Experimental protocols

Rats were acclimatized for one week and randomly divided into 6 groups:

Group 1: Sham + vehicle 24 h;
Group 2: Sham + NR 24 h;
Group 3: IRI + vehicle 24 h;
Group 4: IRI + NR 24 h;
Group 5: IRI + vehicle 14 d;
Group 6: IRI+ NR 14 d.

Each group comprised of 6–7 rats. Models 1–4 had AKI for a short time of 24 hours post-reperfusion, and then the kidneys were removed, while in models 5–6, the kidneys were removed 14 days after IRI.

AKI was induced by bilateral IRI through abdominal incision during anesthesia with hypnorm/ midazolam 5mg/ml, 200 μl/100g rat subcutaneously (Panum Institute, Copenhagen, Denmark). The right and left renal arteries were clamped with arterial clips for ischemia for 45 minutes. Carprofen 5mg/ml, 100 μl/100 g rat subcutaneously (Rimadyl, from Pfizer, Copenhagen, Denmark) was used as pain relief at the surgical procedures and given for the following 3 days. The rats were under daily supervision by the researchers and the animal caretakers from Department of Experimental Medicine, The Panum Institute, University of Copenhagen, and animals were scheduled to be sacrificed if failure to thrive was noted or a weight loss of 20% was observed, fortunately none had to be sacrificed. Rats were randomly selected for sham or IRI. Sham animals went through laparotomy of the same duration and manual manipulation of the kidney without arterial clamping.

### Treatment with Nicotinamide Riboside (NR)

Sham and IRI were further divided into two subgroups, treated with NR (Cat No: S2935, Selleckchem, USA) or vehicle (water). NR administration was performed by oral gavage of 500 g NR per kg rat per day. The dose was chosen according to previous studies [81,82] and pilot studies in our lab. The treatment with NR and vehicle started two weeks before the surgery performance and continued into one day after the surgery in all groups.

At sacrifice, under anesthesia, blood samples were obtained, and the kidneys were harvested. One kidney was instantly snap-frozen in liquid nitrogen and stored at -80°C for further processing, while the other kidney was kept in formalin buffer for histological examination.

### Biochemistry

One mL of blood was drawn into heparinized tubes, centrifuged and plasma was separated, divided into several tubes (to avoid freeze-thaw cycles) and stored at -80°C until analysis. Plasma creatinine, phosphate and total calcium were measured by Vitros Chemistry Analyzer (Ortho-Clinical Diagnosis, Rochester, NY).

### Kidney histopathology

The kidney was fixed in formalin buffer 10% at 4°C overnight flashed and stored in 70% ethanol. The fixed tissue was paraffin-embedded and cut in sections of 3 μm for hematoxylin and eosin (H&E), Periodic acid-Schiff (PAS) stain, or trichrome staining, respectively. The samples were evaluated by a pathologist blinded to the experimental protocol using a Zeiss light microscope (Carl Zeiss micro-imaging). Histological damage was assessed and quantified using the EGTI scoring system evaluating 4 individual components, endothelial, glomerular, tubular and interstitial [80].

### NAD^+^

The level of NAD+ in kidney was measured using a commercial NAD^+^/NADH assay kit (#ab65348, Abcam) and following the manufacturer’s protocol. The NAD^+^ Cycling Enzyme Mix in the kit recognized NADH and NAD^+^, but not NADP or NADPH in an enzyme cycling reaction. Protein concentration was determined using a PierceTM BCA protein assay kit (23225; Thermo Fisher). The content of NAD^+^ was normalized to 100 μg protein content.

### Mitochondria

The relative levels of oxidative phosphorylation (OXPHOS) complexes (I-V) were determined by using an assembly dependent total OXPHOS rodent antibody cocktail (ab110413, Abcam). The kit was used for Western Blotting analysis of the relative levels of the 5 OXPHOS complexes in mitochondria. The samples were heated to 50°C according to the manufacturer’s protocol for denaturalization of the protein.

### Quantitative RT-PCR

The kidney was manually grounded by mortar and pestle, immersed in liquid nitrogen. Total RNA was extracted from the tissue powder using the EZNA RNA kit (Omega Bio-Tek, GA, USA). cDNA was synthesized from RNA with the Superscript III cDNA kit (Invitrogen, MA, USA). Jumpstart (Sigma-Aldrich, MO, USA) and Light cycler 480II (Roche, Basel, Switzerland) were used for qRT-PCR. The mRNA levels were normalized to the mean of three reference genes (S1 Table), which were selected according to the stability using GeNorm software [105].

### Western blot

Protein was extracted from the kidney in T-PER (Thermo Scientific, Rockford, IL) with Halt protease and phosphatase inhibitor cocktail (Thermo Scientific). The protein concentration was determined by the BCA assay (Thermo Scientific). Twenty micrograms of the protein were used for Western Blotting. Blots were visualized by the Amersham ECL Prime Detection Reagent (GE Healthcare, Freiburg, Germany) using the Chemidoc XRS+ System (Bio-Rad). Western Blot quantifications were performed with ImageJ. The following primary antibodies were used in this study to detect: Klotho (ab154163, Abcam), Actin (A5060, Sigma-Aldrich), PARK7 (ab18257, Abcam), LC3B (NB 100–2220, Novusbio), P62/ SQSTM1 (D-3, sc-28359, Santa Cruz Biotechnology, INC.)

### Statistical analyses

Data are presented as mean ± standard deviation (SD). The normal distribution of data was assessed, and calculations were performed in GraphPad Prism 8.0. Statistical significance was tested using an unpaired two-tailed t-test. Significance level set at p ≤ 0.05.

### Ethics

The experiments were performed in accordance with the Danish National Institute for Health’s Guidelines for the Care and Use of Laboratory Animals. The animal models used in this investigation were approved by the Animal Experiments Inspectorate in Denmark (Reference no. 2019-15-0201-01637).

## Supporting information

S1 Fig Effect of IRI on autophagy in the kidney. Western blot analysis and quantification of P62 protein, a marker for autophagy, showed that IRI causes an increase in the level of P62. A significant increase in P62 level was found in the IRI+vehicle compared to the sham+veh group after 24 hours (**A** and **B**). The ratio between LC3BII/LC3BI was unchanged in all groups. Mean ± SD; * p < 0.05.
(TIF)

S2 Fig Inflammation markers, cytokines and pro-inflammatory M1/anti-inflammatory macrophage M2 profile markers in the kidney post IRI. **(A)** The expression of the anti-inflammatory macrophage marker M2, *CD206*, was increased in the NR group at 24 post IRI. **(B)** The expression of the inflammation marker, *CCR2*, was also severely increased in the kidney 24 hours post IRI and still elevated after 14 days with no effect of NR. **(C, D)** The expression of the pro-inflammatory macrophage M1 markers, *CD38* and cytokine *IL6* increased in the kidney tissue at 24 hours and 14 days post IRI. These results indicate together with induction of activin A (as shown in Fig 8), which is involved in M1 polarization state and the decrease in *BMP7*, which is involved in the M2 polarization state, that the classically activated macrophage M1 is pre-dominant in IRI and independent of NAD+ boosting. Mean ± SD; *p <0.05, **p <0.001, ***p <0.0001.
(TIF)

S3 Fig Effect of NAD^+^ boosting by NR on the mitochondrial transcription factor (*Tfam*) in the kidney. The *Tfam* expression in the kidney was significantly upregulated after 24 hours in the sham+NR group, compared to the sham+vehicle group. However, *Tfam* levels remined unchanged in post-IRI rats at 24-hours and 14-days, compared to the sham groups. *Tfam*: transcription factor A. Mean ± SD; ** p < .001.
(TIF)

S4 Fig Uncropped Western blots (WB). **(A)** WB from Fig 4A, (**B**) WB from Fig 4C, (**C**) WB from Fig 5A, the membrane was cut and stripped for incubation with PARK 7, (**D**) WB from Fig 6A, (**E**) WB from Fig 6C, (**F**) WB from S2A Fig.
(TIF)

S1 Table Primer sequences for RT-qPCR. (TIF)
