# Supplementary figures and images for: Effect of NAD+ boosting on kidney ischemia-reperfusion injury
Source: PLoS One. 2021 Jun 1;16(6):e0252554. doi: 10.1371/journal.pone.0252554 (PMC8168908; doi:10.1371/journal.pone.0252554)

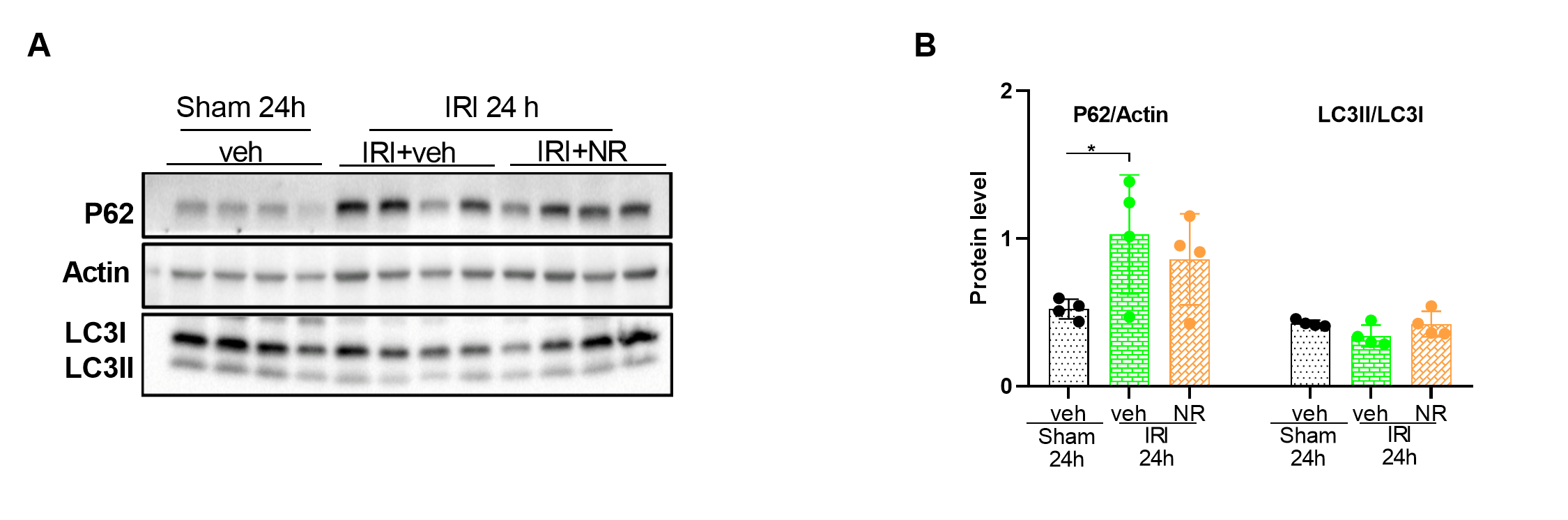

Supplement: S1 Fig — Western blot analysis and quantification of P62 protein, a marker for autophagy, showed that IRI causes an increase in the level of P62. A significant increase in P62 level was found in the IRI+vehicle compared to the sham+veh group after 24 hours (A and B). The ratio between LC3BII/LC3BI was unchanged in all groups. Mean ± SD; * p < 0.05. (TIF) [file pone.0252554.s001.tif]

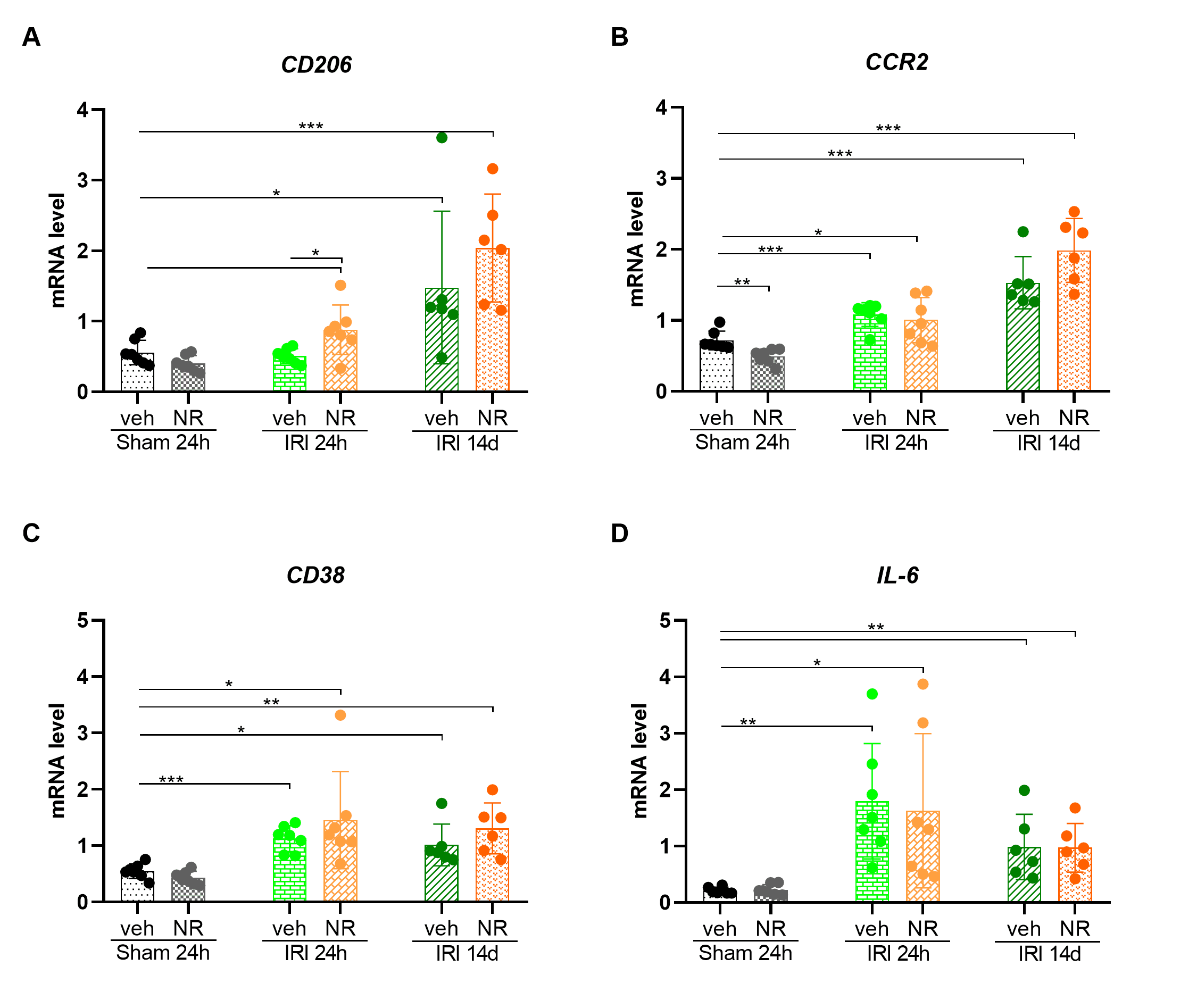

Supplement: S2 Fig — (A) The expression of the anti-inflammatory macrophage marker M2, CD206, was increased in the NR group at 24 post IRI. (B) The expression of the inflammation marker, CCR2, was also severely increased in the kidney 24 hours post IRI and still elevated after 14 days with no effect of NR. (C, D) The expression of the pro-inflammatory macrophage M1 markers, CD38 and cytokine IL6 increased in the kidney tissue at 24 hours and 14 days post IRI. These results indicate together with induction of activin A (as shown in Fig 8), which is involved in M1 polarization state and the decrease in BMP7, which is involved in the M2 polarization state, that the classically activated macrophage M1 is pre-dominant in IRI and independent of NAD+ boosting. Mean ± SD; *p <0.05, **p <0.001, ***p <0.0001. (TIF) [file pone.0252554.s002.tif]

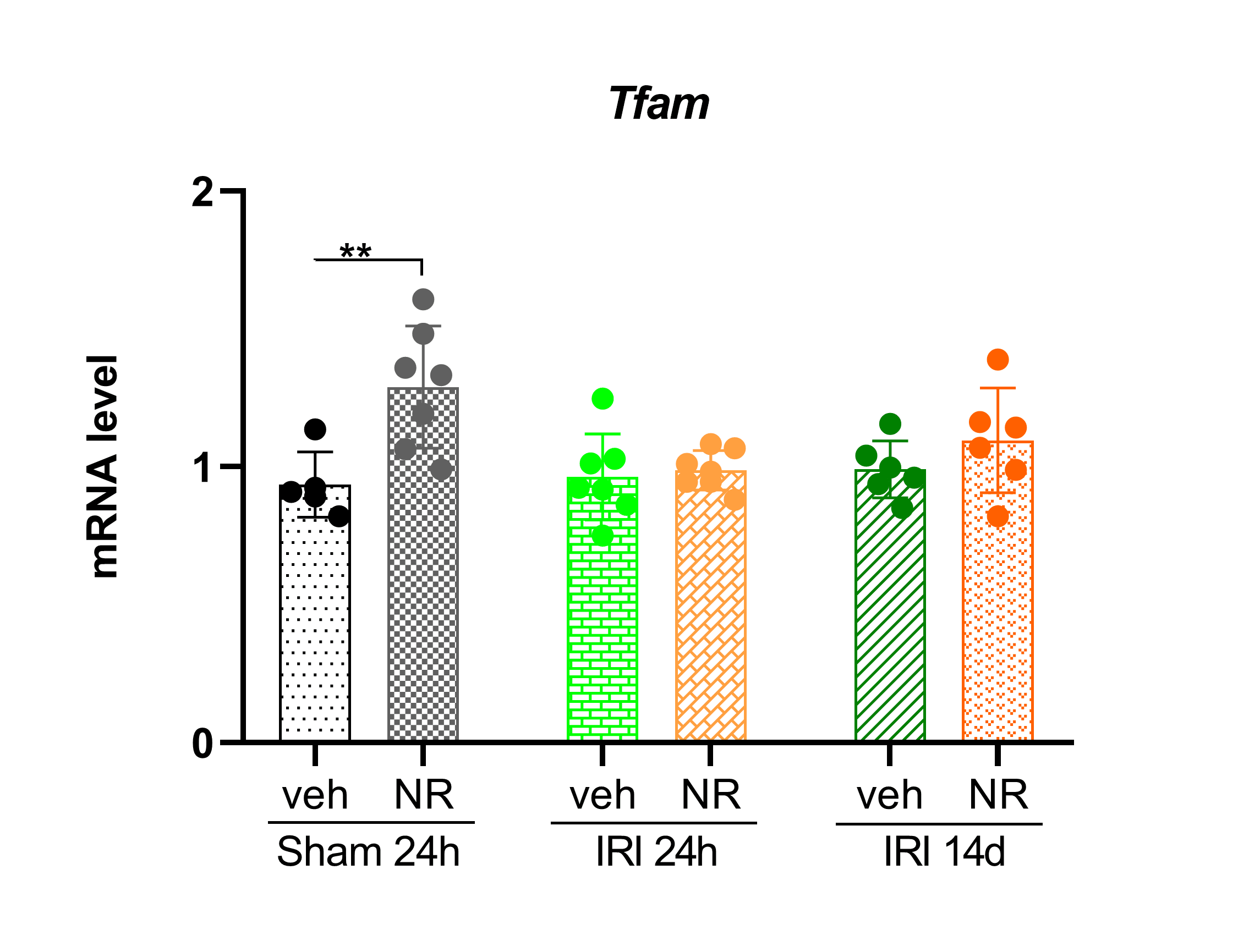

Supplement: S3 Fig — The Tfam expression in the kidney was significantly upregulated after 24 hours in the sham+NR group, compared to the sham+vehicle group. However, Tfam levels remined unchanged in post-IRI rats at 24-hours and 14-days, compared to the sham groups. Tfam: transcription factor A. Mean ± SD; ** p < .001. (TIF) [file pone.0252554.s003.tif]

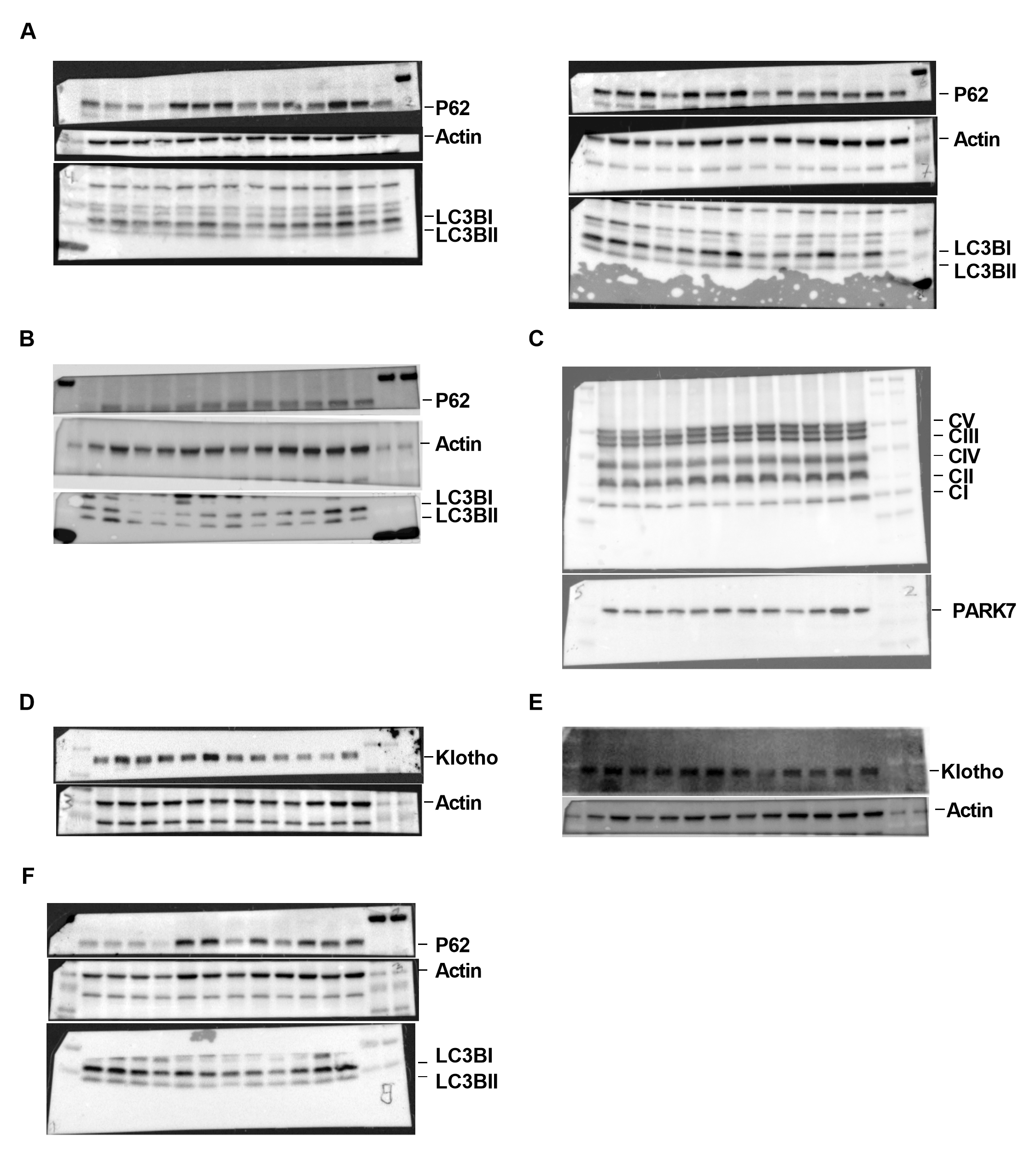

Supplement: S4 Fig — (A) WB from Fig 4A, (B) WB from Fig 4C, (C) WB from Fig 5A, the membrane was cut and stripped for incubation with PARK 7, (D) WB from Fig 6A, (E) WB from Fig 6C, (F) WB from S2A Fig. (TIF) [file pone.0252554.s004.tif]

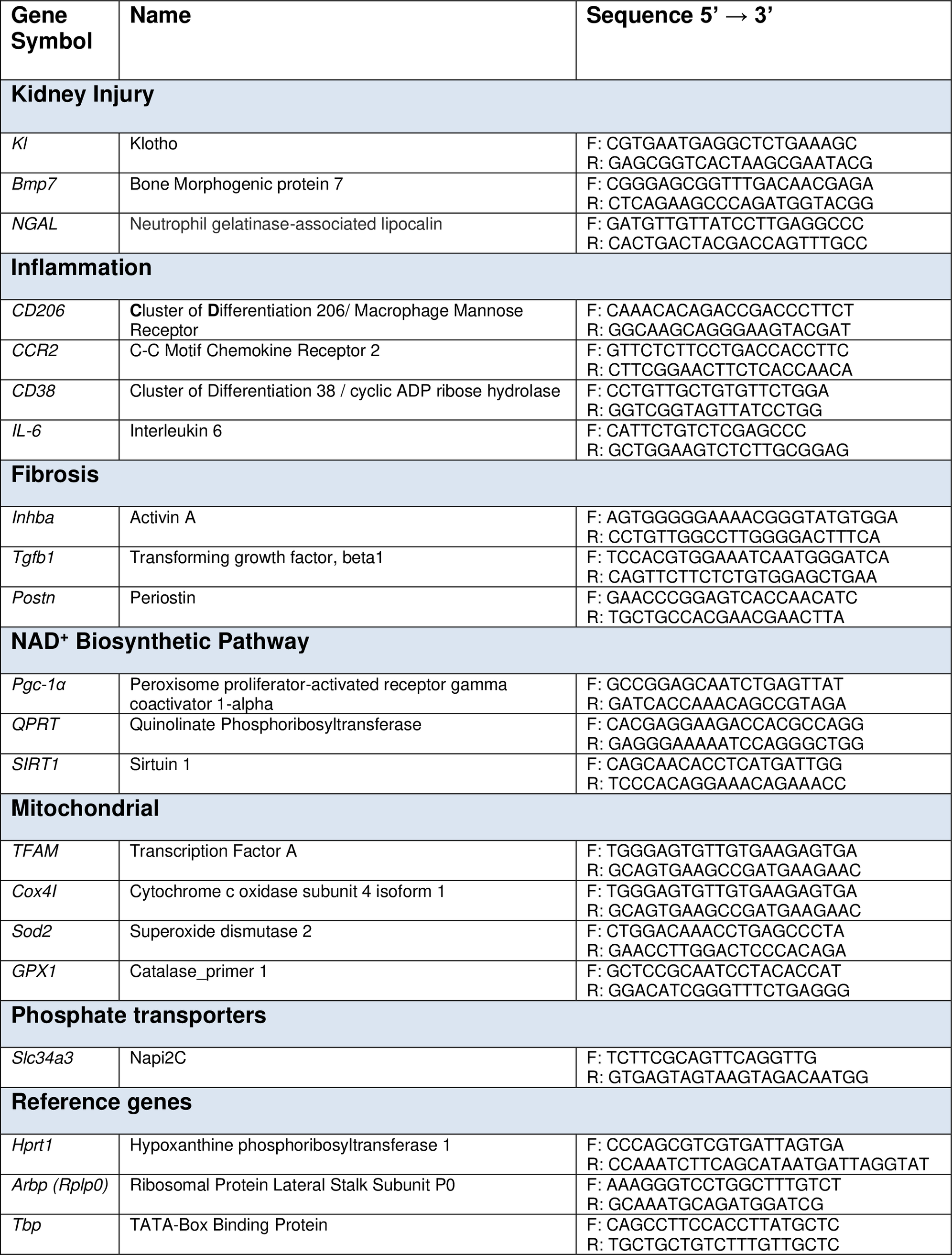

Supplement: S1 Table — (TIF) [file pone.0252554.s005.tif]
